# Corticosterone oscillations during mania induction in the lateral hypothalamic kindled rat—Experimental observations and mathematical modeling

**DOI:** 10.1371/journal.pone.0177551

**Published:** 2017-05-18

**Authors:** Osama A. Abulseoud, Man Choi Ho, Doo-Sup Choi, Ana Stanojević, Željko Čupić, Ljiljana Kolar-Anić, Vladana Vukojević

**Affiliations:** 1 Department of Psychiatry and Psychology; Mayo Clinic, Rochester, Minnesota, United States of America; 2 Chemistry and Drug Metabolism, IRP, National Institute on Drug Abuse, National Institutes of Health, Baltimore, Maryland, United States of America; 3 Department of Molecular Pharmacology & Experimental Therapeutics, Mayo Clinic, Rochester, Minnesota, United States of America; 4 University of Belgrade, Faculty of Physical Chemistry, Studentski trg 12–16, Belgrade, Serbia; 5 University of Belgrade, Institute of Chemistry, Technology and Metallurgy, Department of Catalysis and Chemical Engineering, Njegoševa 12, Belgrade, Serbia; 6 Karolinska Institute, Department of Clinical Neuroscience, Center for Molecular Medicine CMM L8:01, Stockholm, Sweden; College de France, FRANCE

## Abstract

Changes in the hypothalamic-pituitary-adrenal (HPA) axis activity constitute a key component of bipolar mania, but the extent and nature of these alterations are not fully understood. We use here the lateral hypothalamic-kindled (LHK) rat model to deliberately induce an acute manic-like episode and measure serum corticosterone concentrations to assess changes in HPA axis activity. A mathematical model is developed to succinctly describe the entwined biochemical transformations that underlay the HPA axis and emulate by numerical simulations the considerable increase in serum corticosterone concentration induced by LHK. Synergistic combination of the LHK rat model and dynamical systems theory allows us to quantitatively characterize changes in HPA axis activity under controlled induction of acute manic-like states and provides a framework to study *in silico* how the dynamic integration of neurochemical transformations underlying the HPA axis is disrupted in these states.

## Introduction

Bipolar disorder type I (BPI) is a serious medical condition characterized by mania alone or mania alternating with depression. As many as 1% of the general population are estimated to suffer from BPI at some point during their lifetime [1]. The high medical comorbidity [2], suicide rate [3] and economic burden [4] of BPI is accentuated by marginally effective pharmacological therapies [5]. Understanding the neurobiological basis of mania is necessary to develop valid biological markers and create novel therapeutics for bipolar disorder.

Accumulating evidence over the past five decades documents dysregulation of the hypothalamic-pituitary-adrenal (HPA) axis during mania [reviewed recently [6–8]]. However the results of this rich literature remain conflicting. Numerous studies show clear hyperactivity of the HPA axis in manic or BP patients as evident by increased evening cortisol concentrations during mania in plasma [9–15], saliva [16,17] and cerebrospinal fluid (CSF) [18–20]; and high hair cortisol concentrations in BP patients with an illness onset ≥ 30 [21]. More evidence is obtained from studies employing the dexamethasone suppression test (DexST) or the dexamethasone/corticotrophin releasing hormone (Dex/CRH) suppression test to probe the HPA axis. Bipolar manic patients show non-suppression in many well designed studies [9,18,19,22–34]. Along the same lines of HPA axis hyperactivity, recent data showed reduced glucocorticoid receptor responsiveness [35], and increased activity of cortisol metabolizing enzymes 5β reductase and 11β-hydroxysteroid dehydrogenase and a trend towards increased activity for the 5ɑ reductase [36] in bipolar patients. Other studies, however, showed no HPA axis hyperactivity during mania as evident by normal concentrations of urinary tetrahydrocortisol [37], or even lower urinary free cortisol during manic episodes in a rapid cycling patient [38] or lower urinary 17-ketosteroid levels during mania [39–41]. Similar negative findings showed no differences between manic and healthy controls in plasma hydrocortisone [42], plasma 11-hydroxy corticosteroid [43], or plasma cortisol [10,43–46], and even significantly lower plasma cortisol concentrations during manic episodes in two rapid cycling bipolar patients [47]. In addition, measurements of CSF cortisol concentrations between manic and control probands with neurological disorders [48], or controls with severe axis II disorders [49], or healthy controls [50] did not reveal any significant differences. Moreover, normal cortisol suppressions in the DexST were repeatedly observed in manic patients [51–54] and no difference in cortisol levels following Dex/CRH suppression test during manic episodes and during remission was reported in 5 rapid cycling BP patients [55].

These apparently conflicting findings could, to some extent, be attributed to methodological variables such as the timing of sample collection. For example, in Schlesser’s cohort of 61 manic patients where all patients were found to have normal cortisol suppression in the DexST, the plasma cortisol concentration was measured in the morning [51]. In contrast, in studies showing non-suppression cortisol was measured in the evening [23,26]. Also the variability in mania phenotype can lead to conflicting results–for instance, several studies showed HPA hyperactivity is more frequently seen in mixed than pure mania [6,19,20,24,25,52]. Sex differences in the HPA axis in general [56,57] and in manic patients specifically [58] could also contribute to the variability in the results. Another equally important variable stems from the profound complexity of the HPA axis dynamics, with different patterns of hormonal oscillations that require sophisticated ways of data analysis [59].

The aim of this study is twofold: (1) to characterize changes in the HPA axis dynamics during mania-like state induction in the LHK rat model by measuring changes in serum corticosterone levels; and (2) to develop a mathematical model that can emulate these changes.

The LHK rat model is a recently developed animal model where manic-like behavior is induced under controlled conditions by repeatedly stimulating the lateral hypothalamic area with electrical pulses of gradually increasing intensities [60,61]. LHK induces multifaceted manifestations, such as sexual self-stimulation, excessive rearing, feeding and grooming behaviors, and increased total locomotor activity. In this respect, the LHK rat model is more closely reminiscent of human manic episode than the amphetamine-injected rodent model [62] or the Clock mutant mouse model [63], which do not reflect to the same extent the multifaceted symptomatology of mania, and where locomotor activity remains the primary measure of altered behavior.

Mathematical modeling of HPA axis dynamics is a powerful tool to describe the dynamic integration of the nervous and the endocrine systems’ functions, investigate self-regulation of the neuroendocrine system and the effect of internal and external stressors on its activity [64–71]. We have recently developed a five-dimensional reaction model of human HPA axis, with concentrations of corticotrophin releasing hormone ([CRH]), adrenocorticotrophic hormone ([ACTH]), cortisol ([CORT]), aldosterone ([ALDO]) and cholesterol ([CHOL]) as dynamic variables [69,70]. This core model was systematically fine-tuned by Stoichiometric Network Analysis (SNA), a general flux balance analysis method for optimization of reaction networks and determination of instability regions [72–75]. Based on this work, an extended model was developed to describe ethanol effects on the HPA axis [71]. These models were used here as a basis for the development of a rodent model of HPA axis, as described in the Material and Methods section. Mathematical modeling and dynamical systems theory enable us to investigate *in silico* how the underlying biochemical pathways are intertwined to give an integral HPA axis response at the organism level and examine theoretically how dynamic properties of the HPA axis change when the rates of individual pathways change, thereby helping us to understand how HPA axis activity is changed at the organism level in acute or chronic manic states.

## Materials and methods

### Animals and ethics

Experimental protocols were reviewed and approved by the Mayo Clinic Institutional Animal Care and Use Committee and the methods were carried out in accordance with the approved guidelines. Male Wister rats, 250–300 g (Charles Rivers, International, Inc. Wilmington, MA) were housed in individual cages with ordinary bedding under 12 hour light/12 hour dark cycle with lights on at 6:00 AM, temperature was kept around 21°C, and humidity between 40–70% with free access to standard rodent chow and water. Three experimental groups were used: control group (no electrodes implanted in the lateral hypothalamus; n = 6), sham group (electrodes implanted in the lateral hypothalamus but not kindled; n = 7), and kindled group (electrodes implanted in the lateral hypothalamus and kindled; n = 7). During the experimental procedure, ten animals were excluded due to difficult access or blocked jugular vein catheter (n = 4), pulling stimulating electrode and/or jugular vein catheter during mania induction (n = 5) or dying during blood sampling (n = 1) among twenty animals used in this experiment. Sampling was successfully achieved in 3–4 animals/group. The experiments were conducted in three series, totally lasting over three months. In each series, two animals from the control group were analyzed during two consecutive days; followed by analysis of two animals from the sham group and two animals from the LHK group. Thereafter, a new series of experiments was initiated.

### External jugular vein cannulation surgery

Anesthetized animals (isoflurane inhalation: 3.0% during induction and 1%-1.5% during maintenance) were placed on the surgery table and a 2 cm × 4 cm area was shaved on the ventral aspect of the neck, and another 1 cm × 1 cm area was shaved on the upper back of the animal between the two scapulae to externalize the catheter. A 1.5 cm longitudinal skin incision was made ventrally along the midline of the neck. The right external jugular vein was separated from the surrounding tissues. A 5.0 F silastic silicon catheter (#62999–133, inner/outer diameters 0.76/1.65 mm, VWR, IL, USA) filled with heparinized saline (1 unit in 0.1 ml) was inserted into the vein, then secured in place and tunneled under the skin through a subcutaneously implanted skin button with Dacron patch and silicon sleeve (#SBD-02; SAI-Infusion Technologies, IL, USA) to prevent the catheter from slipping during the mania-induction experiments. The catheter was then externalized through the upper back incision and flushed immediately with heparinized saline then sealed with a stainless steel plug. The skin incisions were then sutured with 4/0 silk sutures [76].

### Surgical implantation of stimulating electrodes

Anesthetized animals (isoflurane inhalation: 3.0% during induction and 1%-1.5% during maintenance) were secured in the stereotaxic apparatus (David Kopf Instruments, Tujunga, CA) and the skull levelled between bregma and lambda. Bipolar stimulating electrodes (#MS303 twisted stainless steel, outer diameter 125 μm, Plastics One, Inc., Roanoke, VA. USA) were implanted bilaterally into the lateral hypothalamic area (A/P: -2.28, M/L: ±2.7, D/V: −8.5 mm from skull surface). Electrodes were implanted with a 7° angle to allow enough room to attach the stimulating cords on both sides. Electrodes were secured to the skull using dental cement and three screws. Animals were closely observed during the immediate post-operative interval. Buprenorphine 0.05 mg/kg was administered subcutaneously to alleviate pain and suffering pre and postoperatively. Animals showing any signs of infection (*i*.*e*. swelling, or discharge at scalp incision site) or manifestations of postoperative neurological damage (*i*.*e*. limping, posturing, or inability to move freely) due to electrode implantation were euthanized for humane purposes.

### Mania-induction protocol

Following one hour habituation, bilateral lateral hypothalamic kindling was performed using a 10 s long sequence of square bipolar pulses of 180 Hz frequency and 200 μs pulse width, followed by 30 s of rest. Seven trains were applied, each consisting of 10 pairings of 10 s duration stimulation pulses alternating with 30 s of rest, and 2 min of rest were allowed between trains. Stimulation amplitude, *i*.*e*. electric potential difference between the electrodes was 1 V at the beginning, and was increased by 1 V increments to 7 V for the rest of stimulation trains. Kindling elicited typical manic-like behaviors as reported previously [60,61].

### Blood collection protocol

A total of 31 samples (50 μL each) were collected over 24 hours. Blood volume was automatically replaced with an equal volume of heparinized saline after each sample. Total amount of blood drawn throughout the 24 hour experiment was about 1.55 ml or 7.5% of total blood volume [circulating blood volume in laboratory rat ≈ 64 ml/kg or ≈ 21 ml in a rat weighing 300 g, drawing 1.55 ml ≈ 7.5%]. The stress caused by this degree of hypovolemia is considered mild [77]. Blood was collected to a precooled micro tube then centrifuged (3000 *g* × 10 min at 4°C). Collected serum was stored at—20°C until analysis.

In order to confirm that the baseline corticosterone in the kindled animals is not different from sham and control animals before and after kindling, we have performed blood sampling in kindling experiment in two different ways. In one set of experiments, samples 1–23 were collected at 60 min intervals; samples 24–29 were collected at 10 minute intervals during kindling; followed by samples 30 and 31 collected at 30 min intervals after kindling. In the other set of experiments, sample 1 was collected 50 min before kindling; samples 2–7 were collected at 10 min intervals during kindling (or sham stimulation); samples 8–31 were collected at 60 min intervals. We have verified that increased sampling frequency during a short time–at 10 min intervals during 60 min, does not activate the HPA axis on its own. Occasionally, sampling was temporarily discontinued due to difficulties, most often due to blood clothing or air bubbles in the sampling tube, resulting in a loss of data points. However, sampling was continued according to the protocol, as soon as it was technically feasible. Immediately following the last sample collection, animals were deeply anesthetized in a CO_2_ chamber and euthanized by rapid decapitation.

### Serum corticosterone assay protocol

Serum samples were analyzed in duplicates using commercial ELISA kit (# ADI-901-097, Enzo Life Science, NY, USA) according to manufacturer instruction. Inter-assay coefficient of variance is 4.1%.

### Mathematical modeling

#### Development of the stoichiometric network model of HPA axis activity in rodents

**Fig 1:**
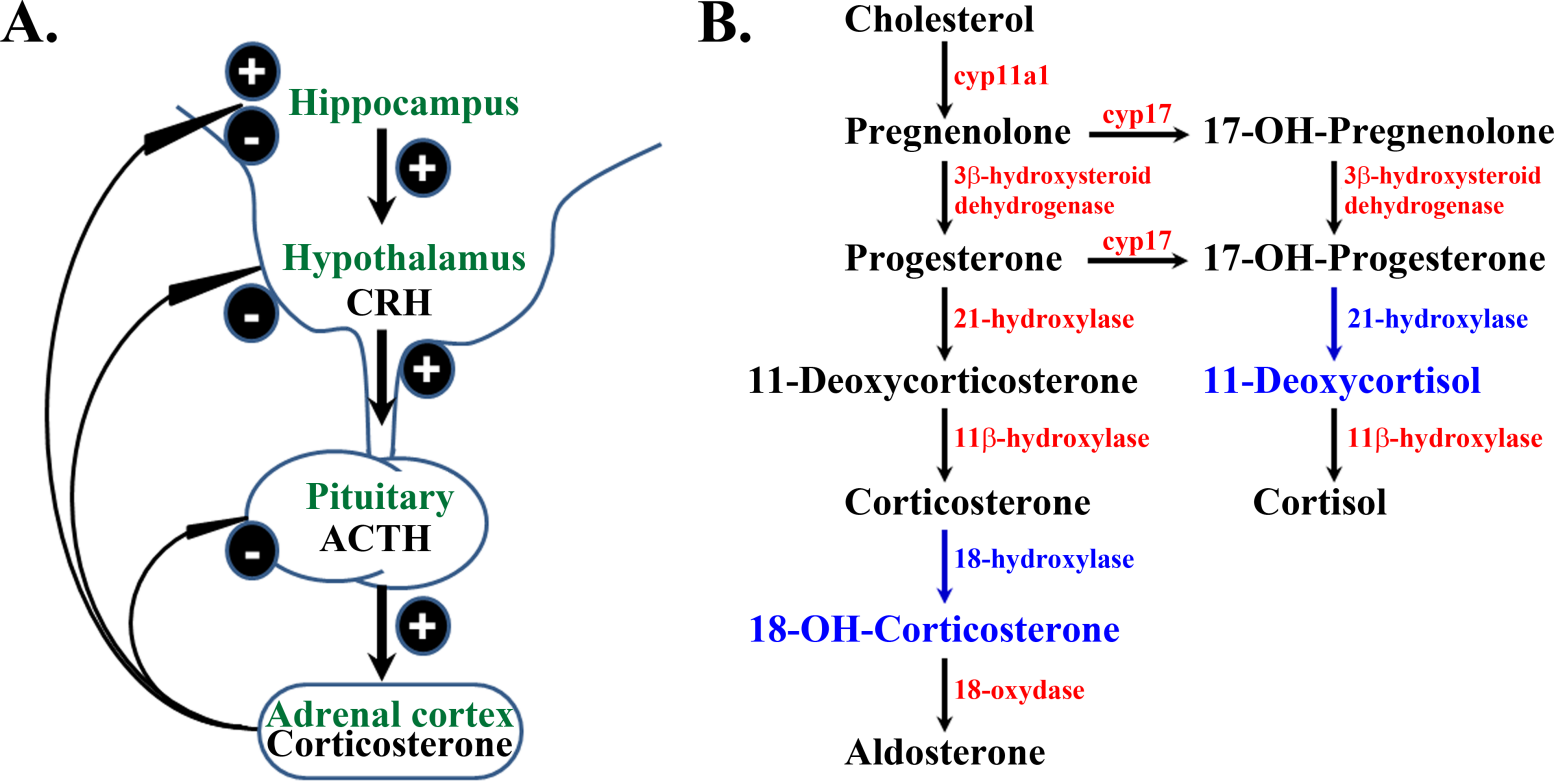
Self-regulation of HPA axis activity. **A.** Concise schematic presentation of biochemical pathways considered in the reaction model of HPA axis dynamics given in Table 1. The reaction model includes CRH, ACTH, ALDO and CORT that comprise the backbone of the HPA axis in humans, and CTS the leading glucocorticoid in rodents. Complex interactions between the considered species give rise to positive (+) and negative (-) feedback loops through which the concentration of all reactive species is finely controlled. **B.** Cholesterol and products of adrenal steroidogenesis included in the reaction model in Table 1 are shown in black. Intermediates that are presently not included in the reaction model are shown in blue. Steroidogenic enzymes that catalyze specific steps in cholesterol conversion to active steroid hormones are indicated in red.

The HPA axis is a complex master integrator of the neuronal and endocrine systems that regulates various bodily processes under basal physiological conditions and stress [78–80]. It synchronizes the actions of the hypothalamus, pituitary and adrenal glands by controlling the plasma levels of corticosteroids secreted from the adrenal glands through a complex cascade of reactions that are intertwined *via* positive and negative feedback loops (Fig 1). HPA axis hormones exhibit complex daily rhythms with two principal periods, ultradian oscillations with a period of 20 min– 2 hours and circadian oscillations with a period of about 24 hours [81–83]. Targeted experimental studies have for many years been unsuccessful in identifying the anatomical origin of ultradian rhythms [84]. However, recent work suggests that an additional level of glucocorticoid autoregulation may exist within the adrenal glands [85], further strengthening the view that birhythmic oscillatory changes in blood glucocorticoid levels reflect the integrated activity of pulsatile hypothalamic forcing on an endogenously rhythmic pituitary-adrenal system.

Based on these experimental findings reported in the literature and building further on our models of HPA axis in humans [64,65,69–71], we developed here a stoichiometric network model that succinctly describes complex neurochemical transformations that constitute the HPA axis in rodents (Table 1). Of note, steps (R1)-(R25) in Table 1 are not elementary biochemical reactions but rather summarized outcomes of complex biological pathways that are concisely presented in the form of stoichiometric relations (see S1 Appendix for detailed description). Consequently, rate constants (k_*i*_) in Table 1 are not experimentally measured kinetic rate constants of any elementary biochemical reaction, but rather values derived by systematic and stringent theoretical analysis of the network of interactions presented in Table 1 (described in the paragraph below). This representation allows us to describe the overall rates of transformation of the neurochemicals included in the model by a set of ordinary differential equations (ODEs) derived based on the law of mass action (S1 Table).

**Table 1:** Stoichiometric network model describing self-regulation of HPA axis hormones in rats.*

Stoichiometric relation			Rate constant	No.
	→	CHOL	k_1_ = 5.5200×10^−5^ M min^-1^	(R1)
	→	CRH	k_2_×*D*; k_2_ = 2.2875×10^−8^ M min^-1^	(R2)
	→	ALDO	k_3_ = 7.3080×10^−11^ M min^-1^	(R3)
CRH	→	ACTH	k_4_ = 1.0811×10^4^ min^-1^	(R4)
ACTH + CHOL	→	PNN	k_5_ = 3.4240×10^7^ M^-1^min^-1^	(R5)
PNN	→	PGS	k_6_ = 7.7371 min^-1^	(R6)
PGS	→	DCTS	k_7_ = 3.8686 min^-1^	(R7)
DCTS	→	CTS	k_8_ = 1.1606×10^−2^ min^-1^	(R8)
CTS	→	ALDO	k_9_ = 1.2000×10^−3^ min^-1^	(R9)
PGS	→	HPGS	k_10_ = 1.0000×10^−5^ min^-1^	(R10)
PNN	→	HPNN	k_11_ = 6.1900×10^−2^ min^-1^	(R11)
HPNN	→	HPGS	k_12_ = 1.2380×10^−2^ min^-1^	(R12)
HPGS	→	CORT	k_13_ = 6.1900×10^−2^ min^-1^	(R13)
DCTS + 2CTS	→	3CTS	k_14_ = 1.5120×10^12^ M^-2^min^-1^	(R14)
ALDO + 2CTS	→	CTS	k_15_ = 8.4600×10^10^ M^-2^min^-1^	(R15)
CRH + CTS	→		k_16_ = 3.6000×10^10^ M^-1^min^-1^	(R16)
ACTH + CTS	→		k_17_ = 3.0000×10^9^ M^-1^min^-1^	(R17)
CHOL	→		k_18_ = 1.6200×10^−1^ min^-1^	(R18)
CRH	→		k_19_ = 6.6000×10^−4^ min^-1^	(R19)
ACTH	→		k_20_ = 6.4200×10^2^ min^-1^	(R20)
CTS	→		k_21_ = 7.3800×10^−2^ min^-1^	(R21)
ALDO	→		k_22_ = 1.6200×10^−1^ min^-1^	(R22)
CORT	→		k_23_ = 1.6200×10^−1^ min^-1^	(R23)
PNN	→		k_24_ = 1.2840×10^−2^ min^-1^	(R24)
PGS	→		k_25_ = 1.0000×10^−5^ min^-1^	(R25)

* Relations (R1)-(R25) concisely represent complex biochemical pathways that constitute the HPA axis. Relations (R1)-(R3) represent the biosynthesis of cholesterol (CHOL), corticotrophin releasing hormone/corticotrophin releasing factor (CRH) and aldosterone (ALDO), respectively. Relation (R4) describes the CRH stimulated adrenocorticotrophic hormone (ACTH) production from the pituitary. Relations (R5)-(R13) concisely summarize the multifaceted synthesis of steroid hormones in the adrenal cortex and their secretion into the global circulation, which starts by ACTH-mediated conversion of cholesterol to pregnenolone (PNN), followed by conversion to progesterone (PGS), deoxycorticosterone (DCTS), 17α-Hydroxypregnenolone (HPNN), 17α-Hydroxyprogesterone (HPGS). Relations (R14) and (R15) describe the positive and negative feedback actions of corticosterone, respectively, which is mediated *via* hippocampal glucocorticoid receptors (GR) and mineralocorticoid receptors (MR). The positive feedback actions of corticosterone are described in the form of cubic autocatalysis (R14), whereas the negative feedback actions of corticosterone (CTS) are described in the form of quadratic autoinhibition with respect to corticosterone (R15). GR and MR concentrations are implicitly included in the kinetic rate constants, and do not appear as independent reactive species in the model. Relations (R16) and (R17) describe the negative feedback action of corticosterone on the hypothalamus and pituitary, respectively, to suppress CRH and ACTH production. Relations (R18)-(R25) describe the removal (by bioconversion and/or elimination) of CHOL, CRH, ACTH, CTS, ALDO, CORT, PNN and PGS from the circulation. Products of bioconversion do not participate as reactants in any reaction and are therefore not specified in the model.

In order to derive rate constants (k_*i*_) for the relations in Table 1, we closely rely on our work that underlies the development of a stoichiometric network model of HPA axis in humans [69–71]. This is possible because the biological pathways that underlie the HPA axis in humans and in rodents and the stoichiometric network of interactions that represents them are structurally similar, *i*.*e*. the number of variables in the system and the way that these variables are connected to each other are analogous. However, the actual values of rate constants (k_*i*_) that define the HPA axis dynamics are not the same in rodents and humans because of interspecies differences and because the leading corticosteroid in rodents and humans are not the same. Derivation of the instability conditions for the 5-dimensional stoichiometric network core model by SNA [72–75] is described in detail in [69,70]. By knowing the instability conditions, the actual concentrations of HPA axis hormones in the peripheral circulation in rats (S2 Table) and the frequency of ultradian oscillations, we could now identify the narrow range of kinetic parameters, *i*.*e*. determine the values of rate constants k_*i*_ that are specific for the HPA axis model in rodents, for which the model in Table 1 yields ultradian oscillations in HPA axis hormone levels that occur in physiologically relevant concentration ranges (S2 Table) and with a realistic ultradian frequency.

#### Numerical simulations

Numerical simulations were performed using MATLAB ode15s solver based on the Gear algorithm for integration of stiff differential equations [86]. Absolute and relative tolerance error values were 3×10^-20^ and 1×10^-14^, respectively. Integration was performed with stricter tolerances in order to minimize potential numerical artefacts, but the same dynamical behavior was observed using values 1×10^−9^ and 3×10^−6^ for absolute and relative tolerance, respectively.

#### Mathematical modeling of HPA axis activity under normal physiology (control conditions)

The reaction model and kinetic rate constants (k_*i*_, *i* = 1–25) used in the numerical simulation of HPA axis dynamics under control conditions are shown in Table 1. Differential equations describing changes in HPA axis hormone concentrations were derived from the biochemical relations given in Table 1 in accordance with the law of mass action (S1 Table). The circadian rhythm was modeled as an asymmetric forcing function, *D*, with a period of 24 hours [69,87,88]:

$$\begin{array}{l} D=d_{1}-0.079145093\times d_{2}+\{(0.064\times \sin(2\pi *(t-840)/1440)+0.12\times abs\left[\sin(\pi (t-840)/1440\right]\}\times d_{2} \end{array}$$

where parameters d_1_ = 0.2662 and d_2_ = 2.5, and 1440 in the denominator of the trigonometric function argument represents the number of minutes in one day, *i*.*e*. in one 24 hours period. The circadian rhythm function *D* is coupled through the CRH production step (R2), and transforms the kinetic rate constant k_2_ into a periodic function k_2_ × *D*. Initial concentrations for integration of the underlying set of ordinary differential equations (ODEs) were the same in all numerical simulations: [ACTH]_0_ = 8.00×10^−13^ M, [ALDO]_0_ = 1.50×10^−9^ M, [CHOL]_0_ = 3.40 ×10^−4^ M, [CTS]_0_ = 4.00×10^−8^ M, [CRH]_0_ = 1.00×10^−12^ M, [CORT]_0_ = 1.50×10^−9^ M, [DCTS]_0_ = 4.00×10^−9^ M, [HPNN]_0_ = 1.00×10^−10^ M, [HPGS]_0_ = 4.00×10^−8^ M, [PNN]_0_ = 1.00×10^−10^ M, [PGS]_0_ = 4.00×10^−8^ M.

#### Mathematical modeling under sham conditions

In order to account for the increased HPA axis activity under sham conditions, the rate of step (R2) describing CRH production was increased by multiplying the rate constant k_2_, *i*.*e*. the value given in Table 1, by 1.1765.

#### Mathematical modeling under acute mania-induction conditions

To model the temporal evolution of corticosterone under LHK, kinetic parameters used for modeling sham conditions were applied. Based on the classical *in vitro* study by Bradbury *et al*. who showed that electrical stimulation of hypothalamus causes a frequency dependent CRH output [89], we modeled the effects of electric stimuli applied in the *in vivo* experiments as acute perturbations with CRH. In order to simulate acute perturbations with CRH, numerical integration of the set of ODEs (S1 Table) was stopped at a specified time point, and new initial conditions for the subsequent integration were defined. For the new initial conditions, CRH concentration was increased for an indicated amount, whereas the concentrations of all other species retained the values that they have attained before the numerical integration was stopped [64,71,90]. This procedure has been repeated every 40 s during 50 min to mimic an actual LHK sequence. The amount of CRH used to mimic LHK in numerical simulations, [CRH] = 5×10^−8^ M, is the value that is determined to give rise to corticosterone levels that best agree with experimentally measured values under LHK.

## Results

### LHK induces a sharp transient increase in serum corticosterone concentration

**Fig 2:**
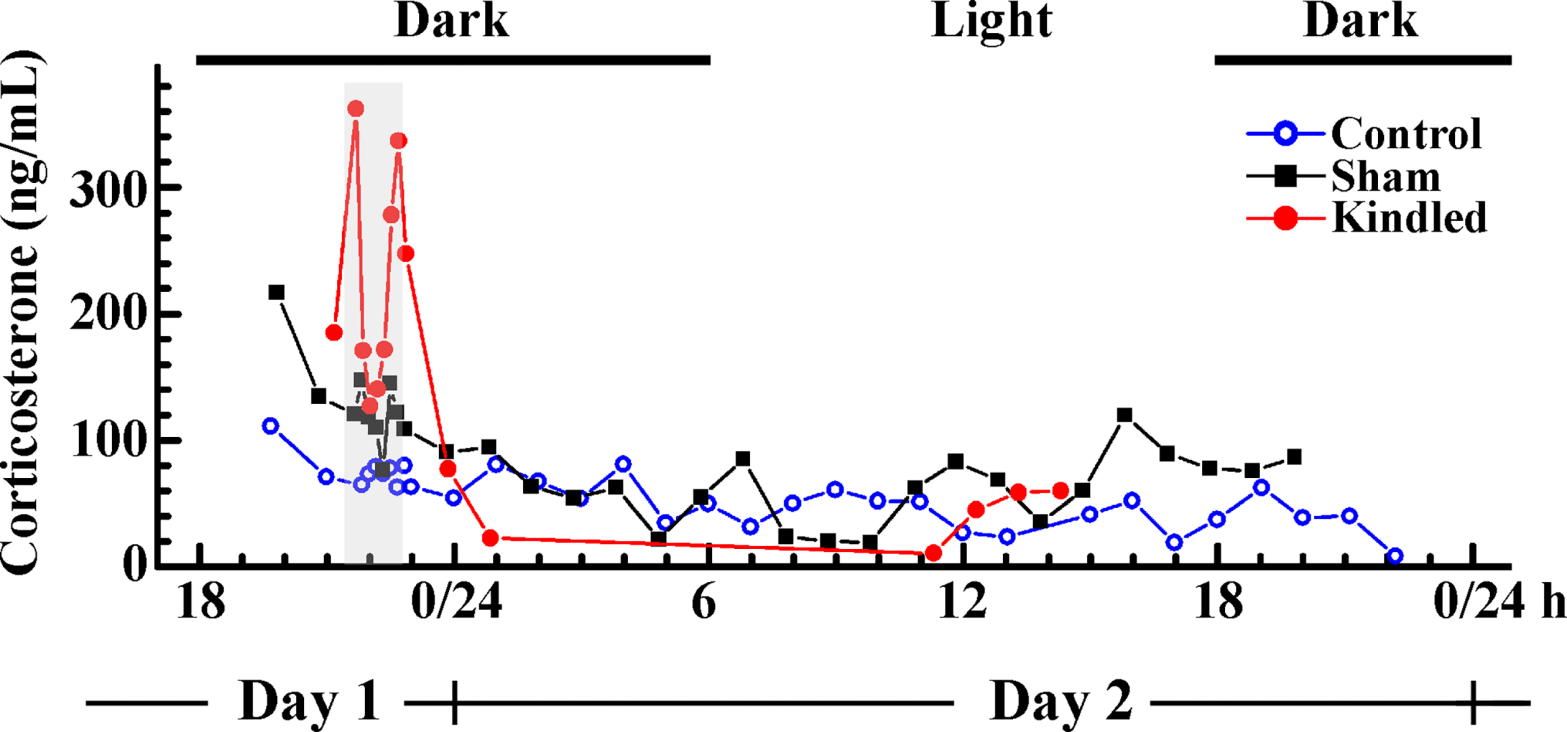
Experimentally measured temporal changes in corticosterone concentrations during LHK. Experimentally measured changes in corticosterone concentrations in individual animals: control (open circle, blue), sham (rectangle, black) and kindled (solid circle, red). The shaded region indicates the interval during which LHK was applied.

**Fig 3:**
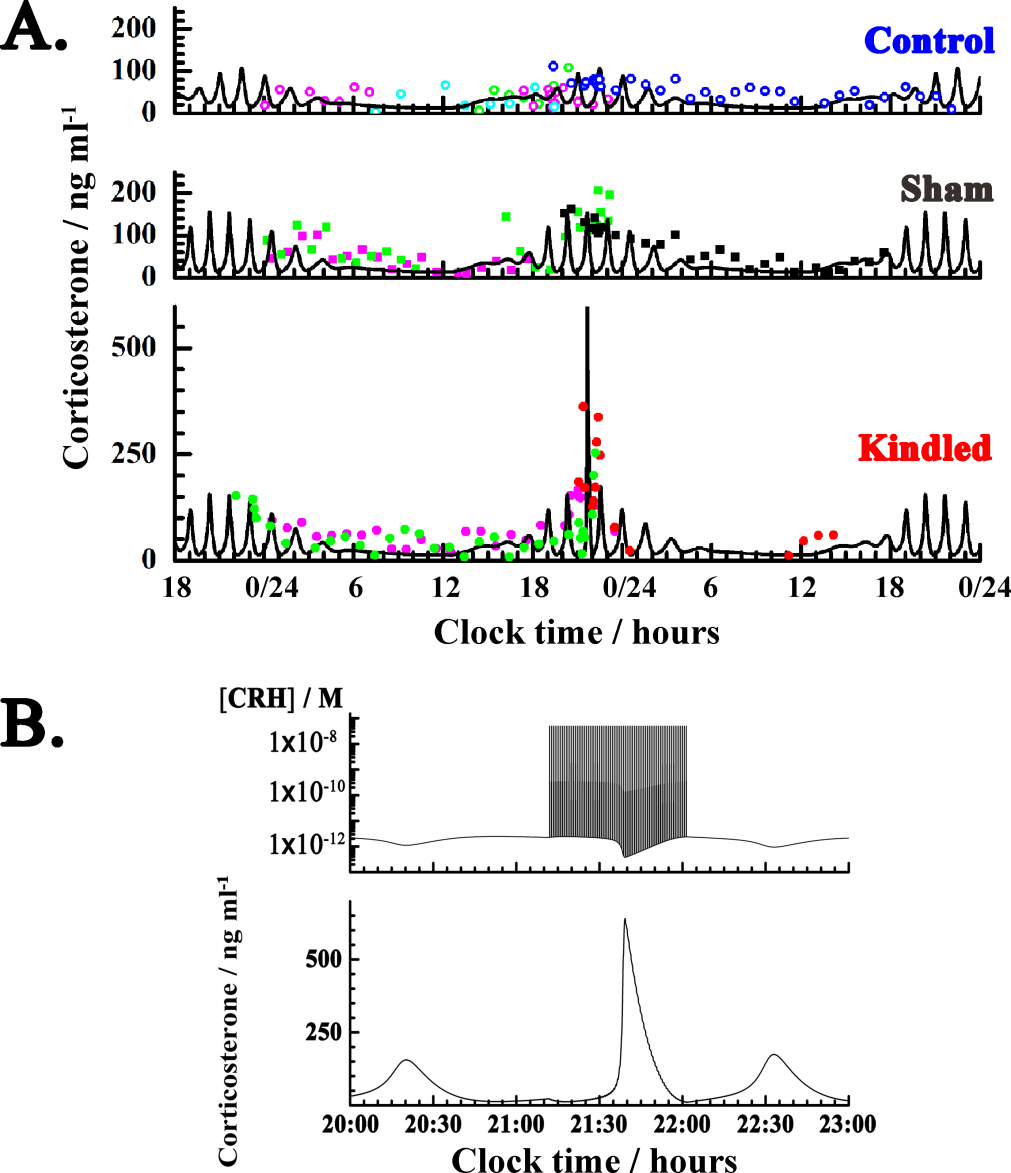
Experimental and numerically simulated temporal changes in corticosterone concentrations during LHK. **A.** Temporal changes in corticosterone concentration under control (top), sham (middle) and LHK (bottom) conditions measured experimentally; n = 3–4 animals/group (symbols) and as predicted by mathematical modeling (solid lines). The data presented with blue circles, black squares and red dots are the same as corresponding data shown in Fig 2. **B.** Time traces showing the dynamic behavior just before, under and shortly after LHK, from 20:00–23:00 h. ***Top*:** Numerically simulated CRH dynamics before, during and shortly after LHK. Individual CRH pulses during an LHK kindling sequence that starts at 21:12 o’clock and lasts until 22:02 o’clock, are visible as vertical lines, representing an abrupt change in CRH concentration induced by a 10 s long sequence of square bipolar pulses of 180 Hz frequency and 200 μs pulse width, followed by 30 s of rest, which was modelled in numerical simulation as an instantaneous perturbation applied every 40 s during 50 min. The intensity of a single CRH pulse was 5×10^−8^ M. ***Bottom*:** Corresponding changes in corticosterone concentrations. This time trace is the same as shown at the bottom (A), except that a shorter time span, from 20:00–23:00 o’clock, is presented here. The first oscillation is the last oscillation before LHK, visible at the bottom of (A). LHK starts at 21:12 o’clock and lasts until 22:02 o’clock. After LHK is discontinued, the HPA axis activity is quickly being restored, as evident from the time trace shown at the bottom of (A).

Experimentally measured changes in corticosterone levels for one representative animal in each group: control, sham and kindled, are shown in Fig 2. Concurrent data sets, consisting of discrete measurements for all animals in a group, are shown in Fig 3 (symbols). One-way ANOVA analysis revealed that overall serum corticosterone concentration increased significantly during LHK (158 ± 16) and sham (128 ± 6), as compared to the control group (72 ± 3) respectively [mean ± s.e.m., ng/mL; F_2,53_ = 14.5, p < 0.001]. However, this effect is transient and disperses quickly after kindling is discontinued (Fig 2).

It is important to underline here that increased blood sampling frequency (10 min *vs* 60 min) does not seem to have any significant effect on the observed potentiation of HPA axis activity, as evident from Fig 3A (bottom panel, green dots), where this effect was assessed. In this experiment the sampling was increased to one sample *per* 10 min during 50 min (around 22 hours) without performing LHK, but obvious HPA axis activation was not observed.

### Mathematical modeling of HPA axis dynamics under normal physiology and under LHK

Numerical integration of ODEs (S1 Table) yields the temporal evolution of daily corticosterone levels under normal physiology (Fig 3A, top; solid line). Here we show data for corticosterone because we have experimental measurements only for corticosterone, but the model predicts the evolution of daily levels of all neurochemicals considered in the model and the agreement between model predictions and experimentally measured HPA axis hormone concentrations is very good (S2 Table).

We show the results of numerical simulation for one set of control parameters in each group (Fig 3A–3C, solid lines) for the following reason. It is well-established that diurnal and ultradian HPA axis dynamics under normal physiology is reasonably stable for an individual but can significantly differ between individuals [91]. Our experimental data do not have sufficient temporal resolution to characterize ultradian oscillations in an individual animal. By presenting the data indiscriminately, taking into account data from all animals in the same group, we could optimize the model and derive one set of control parameters that best agrees with all data from the same group. In reality, one should actually use slightly different parameter values for each animal in order to account for individual differences in circadian and ultradian rhythmicity. However, since our aim here is not to mimic the HPA axis dynamics in an individual animal, but rather to model a general response, this approach is fully justified.

Experimental analysis of temporal changes in corticosterone concentration under control, sham and LHK conditions shows that average corticosterone levels and the amplitude of ultradian corticosterone oscillations increase under acute induction of manic-like states by LHK (Figs 2 and 3). It also shows that these effects are transient, and disperse quickly after kindling is being discontinued (Figs 2 and 3, red dots). Mathematical modeling can veritably imitate these experimental observations and shows that average corticosterone levels, the amplitude and frequency of ultradian corticosterone oscillations all increase under sham conditions and under acute mania-induction (Fig 3, solid line). In addition, mathematical modeling also showed that these effects are transient when the kindling time is short (here 50 min), and disperse quickly after kindling is discontinued (Fig 3A, bottom; magnified in Fig 3B), in agreement with experimental observations (Fig 2).

### Changes in HPA axis dynamics induced by LHK of different intensity and duration predicted by mathematical modeling

**Fig 4:**
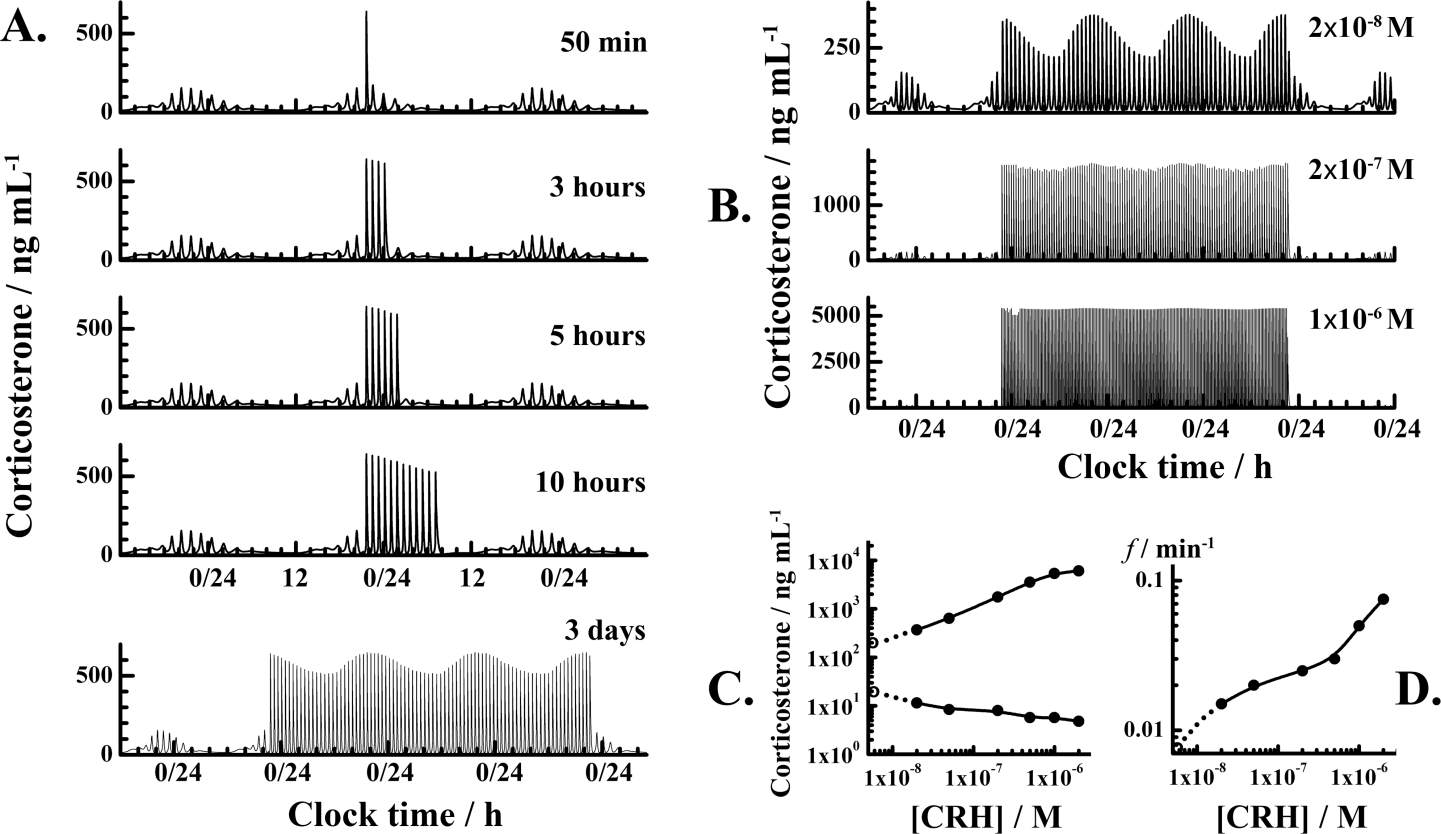
Effect of LHK duration and intensity on HPA axis activity. **A.** Changes in HPA axis dynamics induced by LHK of different duration: 50 min, 3 hours, 5 hours, 10 hours and 72 hours (from top to bottom) but the same intensity of a single CRH pulse, 5×10^−8^ M. **B.** Changes in HPA axis dynamics induced by LHK of the same duration (72 hours) but different intensity. The intensity of a single CRH pulse during LHK was: 2×10^−8^ M (top), 2×10^−7^ M (middle) and 1×10^−6^ M (bottom). **C.** The amplitude of ultradian corticosterone oscillations increases as the intensity of LHK is being increased. Solid circles indicate the highest and the lowest value of the ultradian corticosterone oscillation with the largest amplitude. Open circles indicate the corresponding values under sham conditions, without LHK. **D.** The frequency of ultradian oscillations increases as the intensity of LHK is being increased. The open circle at the origin indicates the frequency of ultradian oscillations under sham conditions, without LHK.

The mathematical model predicts that with continuous kindling the HPA axis will enter a new dynamical regime and will maintain large-amplitude ultradian oscillations as long as kindling persists (Fig 4A). According to the mathematical model predictions, LHK of increasing intensity increases the amplitude and the frequency of ultradian corticosterone oscillations (Fig 4B–4D). This increase in ultradian oscillation amplitude is accompanied by further deviation from the normal oscillatory pattern into complete loss of circadian oscillations (Fig 4B, bottom panel). While further studies are needed to characterize the effect of progressively longer mania-induction durations on oscillatory behavior of serum corticosterone and test the predictive validity of the model, the results in Fig 4B clearly demonstrate HPA axis allostasis, *i*.*e*. the process of achieving stability by changing the underlying dynamics–as the allostatic load increases with increasing LHK intensity, the HPA axis alters its dynamics accordingly (Fig 4B–4D). This adaptation lasts only as long as the HPA axis is under the influence of LHK and normal dynamics is restored after LHK is discontinued (Fig 4A).

## Discussion

The results of this study show that LHK causes robust increase in serum corticosterone, a surrogate marker of HPA axis hyperactivity, which is transient in nature and lasts only as long as LHK is applied (Figs 2 and 3). This effect could be emulated by the mathematical model in Table 1 as a consequence of an increased CRH output caused by LHK (Fig 3B). This novel finding strengthens the notion of an important role of CRH in the pathophysiology of mood disorders [92], and is in agreement with clinical data on hypothalamic hamartoma–patients with this non-neoplastic tumor present with mood symptoms [93] and also elevation in CRH concentration [94]. However, it might seem contradictory to other data of emerging manic symptoms in patients receiving corticosteroid treatment for medical conditions (reviewed in [95]). It is possible to speculate that exogenous corticosteroids also have indirect effect on the hypothalamic neuronal firing. Indeed, Chu et al. [96] showed that exogenous CRH decreases hypothalamic neuronal firing through reducing T-type Ca^2+^ channel activity. In this scenario, treatment with corticosteroids exerts a negative feedback at the level of the hypothalamus suppressing CRH secretion. Low CRH levels could release the suppression of hypothalamic neurons and cause an increase in neuronal firing and excitability. Under stress or in vulnerable individuals, this enhanced excitability could manifest as manic-like symptoms.

Interestingly, our experimental studies presented here show that HPA axis dynamics in the sham group showed slight “potentiation” as compared to the controls (Fig 2). This observation is in agreement with clinical data showing persistently abnormal HPA axis activity in euthymic bipolar patients [16,17,29,31–33,97,98]. Based on the fact that sham animals only had an electrode implanted in the LH bilaterally, it is possible that the surgical intervention, anesthesia [99] and/or stress due to the mere presence of electrodes could be instrumental in changing neuronal excitability, thus affecting serum corticosterone. Further studies are needed to assess these contributions. However mathematical modeling showed that this effect can be mimicked when CRH production is increased *via* the rate constant k_2_, showing that good agreement with measured corticosterone in this group is achieved when the stress factor in the model is increased.

It is important to note that the measured corticosterone concentrations show variability between individual animals in the same group (Fig 3A, symbols). This variability could be explained by the well-established fact that under normal physiological conditions, diurnal and ultradian HPA axis dynamics are stable in a given individual but can significantly differ between individuals [91]. Merging the data within the same group, as was done in this study (Fig 3A), prevents us from detecting individual variations in the estimated model parameters, *i*.*e*. rate constants, but it ensures a good robustness of the values obtained for each group. Because of biological restrictions on blood sampling frequency, we do not have sufficient temporal resolution to characterize ultradian oscillations in individual animals, but Fig 3A clearly shows that the apparent scatter of experimental data points is due to diurnal and ultradian oscillations in their level, rather than due to experimental measurement errors. Thus, we conclude that our mathematical model predicts the complex daily dynamics of HPA axis activity under normal physiological conditions from different aspects. First, the model predicted hormone levels that are, in most cases, in quantitative agreement with published concentrations of HPA axis hormones in rodent models (S2 Table); second, the period of ultradian oscillations is also in good agreement with previously published data acquired with high temporal resolution [100]; third, the model predicts amplitude-dependent increase in CRH values with stimulation which is concordant, despite with different concentrations, with another report showing frequency-dependent CRH concentration with hypothalamic stimulation [89]. In our model, we have chosen the CRH value 5×10^−8^ M based on our measured corticosterone data–so that the level of corticosterone that is “generated” in numerical simulations is of the same order of magnitude as what was experimentally measured. This assumption did not negatively impact the ability of the model to predict HPA axis hormones, and the predicted value is in accordance with *in vitro* experimental studies showing that 1.5×10^−8^ M– 2.50×10^−7^ M CRH modulates voltage-gated ion currents important for the generation of action potentials in CA1 and CA3 pyramidal neurons of rat/mouse hippocampal brain slices [101,102]. We can also compare the model-predicted value with CRH amounts used in a CRH stimulation test, where a CRH dose of 1 μg/kg (humans) or 10 μg/kg (rats) is typically administered as a single intravenous (i.v.) injection, yielding a CRH concentration of 3×10^−9^ M < [CRH] < 3×10^−8^ M in the peripheral blood circulation (calculated for 5.25 l of blood in an average male of 70 kg; or for 70 ml/kg for rats). This level of change in CRH concentration is typically well tolerated, with the most common side effects being transient facial flushing and rare dyspnea or hypotension [103]; and was shown to induce a 3-fold increase in corticosterone concentration [104]. In comparison with these values, an increase in CRH levels of 5×10^−8^ M in an LHK-induced manic-like episode, as was predicted by the model, seems not to be entirely unrealistic, but calls for testing in future studies.

Our work is also of more general bearing. In this study, we used mathematical modeling to explore only few aspects of the complex HPA axis dynamics. However, the reaction model developed here (Table 1) has the capacity to probe other clinically relevant changes, which are beyond the access of previously developed mathematical models of HPA axis activity [66,100,105,106]. For example, the effects of steroidogenesis enzymes activity on HPA axis dynamics shown by several groups to be affected in bipolar patients [36] could be easily probed by modifying the rate constant for corresponding reaction steps in the reaction model, thereby mimicking the effects of enzymatic activity on the HPA axis dynamics. Similarly, the effect of cholesterol, the only precursor of steroid hormones, and of all peptide and steroid hormones that are variables in the reaction model can be systematically examined.

Having said this, we also need to caution about limitations of mathematical modeling of dynamical biochemical systems, in general, and the model presented here, in particular. The HPA axis is an inherently complex dynamical system. It is made up of a large number of different constituents, molecules and cells, which interact to build a spatially and temporally intertwined dynamical network. In this intricate network, molecules produced by one type of cells are distributed across tissue/organ/the whole organism to act on other types of cells, stimulating them to produce other molecules that exert feedback and/or feedforward actions and thus regulate the rates at which biochemical transformations occur in pathways that comprise the HPA axis. As a consequence, the HPA axis acquires a collective feature that is not characteristic of the individual pathways, such as the capacity to self-organize, *i*.*e*. to self-adjust its essential variables in response to signals from within and from the surroundings in order to maintain within acceptable limits its own dynamical structure. When building mathematical models, we substitute this vast complexity by a tractable set of mathematical equations that can imitate these intricate dynamical features. Hence, all mathematical models of complex biochemical systems are an oversimplified representation of the real system and are therefore limited. This is also true for the mathematical model developed here (Table 1), where the effect of a number of important molecules known to affect HPA axis activity, most notably arginine-vasopressin (AVP), angiotensin II (ATII), epinephrine (adrenalin), dopamine, serotonin *etc*., is not considered.

In addition, CRH generation was modeled here using a continuous function (R2), with a rate that varies with the circadian rhythm represented by the periodic function *D*, whereas in reality CRH is not continuously released but is rather discharged in discrete pulses several dozen of times *per* day. While continuous deterministic models of chemical kinetics may give solutions that differ from the ones obtained by models that take into account inherent fluctuations in the concentration of reactants, this difference becomes important under conditions that involve a low number of molecules (*e*.*g*. < 10^4^) or in the proximity of bifurcation points.[107,108] Under other conditions the errors caused by assuming a continuous range of possible concentrations are usually very small, and the continuous and discrete approach yield the same macroscopic steady state solution [107,108]. Hence, continuous inflow of CRH, while clearly a simplification, is not unfounded as long as the average CRH concentration in a pulsatile CRH regime is the same as in the continuous flow regime.

Another important limitation of our model is that many complex processes were combined into one reaction step, such as the reaction steps (R1)-(R25) in Table 1. This conciseness, while a necessary first step towards determination of the overall behavior of the HPA axis, needs to be disentangled if more detailed questions about the role of specific processes, such as the role of gene transcription and translation, protein synthesis, post-translational modification, intracellular trafficking *etc*. are to be examined.

Finally, we underline that all reactive species were treated in our model as if they were spatially homogenous, which is clearly not the case. Once again, this simplification is a justifiable first approximation for the current purposes, where we examine the effect of LHK on the overall dynamics of the HPA axis, but spatial discretization needs to be included if specific questions about the role of different compartments, *e*.*g*. the contribution of different brain regions, want to be addressed.

Having in mind these limitations, the model of HPA axis in rats that is developed here (Table 1), as well as mathematical models of any dynamical biochemical system, should always be regarded as a work in progress and mathematical modeling should be regarded as an iterative process that alternates between laboratory measurements and numerical simulations. Mathematical models are always built based on our current understanding of the problem and on existing experimental data. However, new technologies with improved detection sensitivity continuously provide important new insights. When new experimental facts are obtained, one needs to re-examine whether the existing mathematical model can or cannot account for these new observations and, based on the outcome, decide whether to refine them or to reject them.

Along the same lines, we would also like to point out that while in this study we tested our mathematical model on the experimentally established LHK-induced CRH surfeit [89], further studies are needed to investigate the hypothesis that HPA over-activity in LHK may be driven by an excess of the AVP hormone [15,109]. There is abundant evidence demonstrating the co-expression of CRH and AVP in hypothalamic neurons [110], possibly to potentiate the effects of CRH on pituitary corticotrophes and to coordinate the activation of the HPA axis during chronic stress by driving the release of ACTH [111]. AVP modulates complex social behavior, emotional states, aggression with sensitivity to psychosocial stress [112]. Furthermore, CSF AVP was increased in manic patients compared with depressed, schizophrenic and healthy controls [113] and in bipolar patients taking lithium compared to healthy controls [114], and an association was recently found between the TT genotype of rs28536160 polymorphism of the AVPR1b gene and bipolar disorder with psychotic features and also with genotype CC of rs1293651 polymorphism of CRHR1 gene [115]. At this stage, we have included steroid hormones in our mathematical model and not included AVP in order to keep the model concise and tractable, yet sufficiently resourceful to recapitulate the most important features of the complex real system that we are modeling. This first approximation is justifiable on factual basis–it is well established that CRH is the primary and most potent activator of the HPA axis and that AVP acts primarily as a modifier of its activity, rather than its main driver [116–118]. In addition, there are, to our best knowledge, no experimental data on the daily dynamics of AVP that are acquired with such a high temporal resolution to enable us to distinguish the contribution of CRH from the contribution of AVP and refine our model in accordance with these data. There are also no primary data on the effect of LHK on AVP. Thus, future studies measuring with high temporal resolution AVP and other hormones in LHK are urgently needed.

Similarly, in this study, we used a wide range of stimulation intensity (1V – 7V) and we did not study the effect of a narrow range of stimulation intensity on HPA axis dynamics. We have previously shown that LHK of wide stimulation intensity range (1V – 7V) and the limited stimulation intensity range (1V – 2V) reliably induces manic-like behaviors in male and female rats [60,61]. Male rats seemed to exhibit manic-like behaviors at higher stimulation threshold compared to females, so we adopted in this study the wide range stimulation intensity scale to achieve the behavioral phenotype. Future studies are needed to characterize the relationship between specific stimulation parameters and individually induced manic-like behaviors.

### Concluding remarks

In this study, we have found sharp increase in serum corticosterone during LHK (Fig 2, red) and developed a mathematical model of HPA axis in rats (Table 1) that can emulate these changes by numerical simulations (Fig 3). The observed increase in corticosterone levels is in agreement with a large body of literature showing HPA axis hyperactivity during manic episode. Mathematical modeling predicts that excessive LHK would drive the HPA axis dynamics away from its regular pulsatile oscillation pattern, eventually causing a loss of circadian rhythmicity (Fig 4) and, hence, loss of an important dynamic self-regulation mechanism. Due to lack of experimental evidence, it is presently not possible to assess the accuracy of these predictions, but we demonstrate here how mathematical modeling can be used to provide information for states for which experimental data is scarce.

Our experimental results show for the first time that LHK causes an increase in serum corticosterone concentration in the rat that is reminiscent of mania-associated HPA axis hyperactivity in humans. Further studies are needed to establish whether a “potentiated” HPA axis could serve as a vulnerability marker for predicting the onset/duration of future manic episodes, but the encompassing interdisciplinary approach presented here, by rigorously controlled animal experiments, numerical simulations and dynamical systems theory, represents a significant step forward towards the development of quantitative tools to investigate self-regulation of HPA axis activity in order to understand how the inherently complex neurochemical transformations, through which a coherent HPA axis activity is maintained under normal physiology, are disrupted in manic-like states.

## Supporting information

S1 Appendix Stoichiometric model describing HPA axis dynamics in rats. (DOC)

S1 Table Differential equations describing the temporal dynamics of HPA axis hormones in rodents derived from the reaction model in Table 1 (in the main text). (DOC)

S2 Table Normal basal blood levels of HPA axis hormones in rats as compared to values predicted by the model given in Table 1 (in the main text). (DOC)
