# Supplementary material for: Corticosterone oscillations during mania induction in the lateral hypothalamic kindled rat—Experimental observations and mathematical modeling
Source: PLoS One. 2017 May 18;12(5):e0177551. doi: 10.1371/journal.pone.0177551 (PMC5436765; doi:10.1371/journal.pone.0177551)
Supplement: S1 Appendix — (DOC) [file pone.0177551.s001.doc]

***Supporting Information PONE-D-15-56451***: Abulseoud *et al.* HPA axis dynamics in LHK rat

**S1 Appendix. *Stoichiometric model describing HPA axis dynamics in rats.***

The Hypothalamic-Pituitary-Adrenal (HPA) axis is a dynamic regulatory network of biochemical interactions that integrates and synchronises the nervous and the endocrine systems functions at the organism level. In order to describe how this vast network of interactions operates, we have developed a stoichiometric network model to concisely describe neurochemical transformations that comprise the HPA axis. In a stoichiometric network model of a biochemical system, the outcomes of complex biochemical pathways are concisely described by stoichiometric relations, in an analogous way as elementary chemical reactions are represented by stoichiometric equations. In this representation, substances that initiate, *i.e.* enter a pathway are regarded to behave as reactants; substances that are generated/secreted are regarded to behave as products; and the rates at which reactants/products of a pathway disappear/appear are jointly proportional to the amounts of the reactants. In this way, a mathematical framework is developed to describe in the form of a system of ordinary differential equations (ODEs) the integration of biochemical pathways that constitute the HPA axis on chemical kinetics basis. This, in turn, allows us to use numerical simulations to investigate how the underlying biochemical pathways are intertwined to give an integral HPA axis response at the organism level. Giving that the HPA axis is a nonlinear dynamical network, its response is complex and can often not be intuitively predicted.

In the model presented in Table 1 (in the main text), relations (R1) – (R3) describe the outcome of complex biological pathways that are not part of the HPA axis, but have important consequences on HPA axis function, such as biosynthesis of cholesterol (R1), CRH production from the hypothalamic paraventricular nucleus (R2) and aldosterone production under the renin-angiotensin system control (R3). To account for the circadian regulation of CRH production, which is governed by the circadian clock system in the suprachiasmatic nucleus (SCN) in the anterior hypothalamus, an extrinsic periodic function (*D*) is designed to appropriately mimic the asymmetry of the 24-hour rhythm in rodents:

$\begin{matrix} D = d_{1} - 0.079145093\times d_{2} + \{(0.064\times\sin(2\pi*(t-840)/1440) +0.12\times abs\left[\sin(\pi(t-840)/1440\right]\}\times d_{2} \\ \end{matrix}$where parameters d_1_ = 0.2662 and d_2_ = 2.5, and 1440 in the denominator of the trigonometric function argument represents the number of minutes in one day, *i.e.* in one 24 hours period. The circadian rhythm function *D*, coupled through the CRH production step (R2), transforms the rate constant k_2_ into a periodic function k_2_ × *D*, rendering the intensity of k_2_ in such a way that different values apply at different times of the day. Parameters d_1_ and d_2_ decouple the effects of mean daily CRH level, which is governed by d_1_, from the amplitude of circadian CRH oscillation, which is governed by d_2_. Separation of these parameters in the function *D* enables us to efficiently model individual differences – it is well established that the circadian HPA axis dynamics under basal conditions is very stable for an individual, but can significantly differ between individuals. In the context of our study, this allows us to describe individual differences in daily corticosterone dynamics for each animal (Fig 3A, symbols) by using individually adjusted d_1_ and d_2_ values. However, since the goal of our study was not to focus on individual differences, we have used one parameter set for each group: control, sham and kindled animals, to describe the daily rhythmicity in corticosterone levels (Fig 3A, black line).

Relation (R4) describe the CRH stimulated production of ACTH from the pituitary.

Relations (R5)-(R13) concisely describe the multifaceted biosynthesis of steroid hormones in the adrenal cortex and their secretion into the global circulation. This process, which commences by ACTH binding to the melanocortin receptor type 2 in the middle layer of the adrenal cortex (*zona fasciculata*), is immediately followed by cholesterol mobilization from cellular stores to the inner mitochondrial membrane where cholesterol sidechain cleavage cytochrome P450 (P45011A1) converts cholesterol, the precursor of all steroid hormones, to pregnenolone (PNN); as succinctly described by relation (R5). This immediate response is followed by ACTH-mediated upregulation of transcription of genes encoding steroidogenic enzymes, and steroidogenesis ensues; represented in a simplified way by relations (R6)-(R13) .

Relations (R14)-(R17) describe the complex dynamical regulation of HPA axis activity by glucocorticoids in different forebrain structures, through which they regulate their own production *via* adrenal steroidogenesis. The positive and negative feedback actions of corticosterone mediated *via* GR and MR are described in the form of autocatalysis (R14) and autoinhibition (R15), respectively. GR and MR concentrations are implicitly included in the rate constants and do not appear as independent reactive species in the model. Relations (R16) and (R17) describe the well-established inhibitory effects of glucocorticoids on CRH and ACTH secretion, respectively.

Relations (R18)-(R25) describe the removal of reactive species at a rate that is proportional to their concentration. Removal was explicitly considered only for those species for which the concentration in the model would otherwise not be in the physiological range. Products of bioconversion/elimination do not participate as reactants in any reaction and are therefore not specified in the model.

The corresponding set of ordinary differential equations (ODEs) derived from the model (Table 1, in the main text) in accordance with the law of mass action is given in S1 Table.

**Abbreviations**

ACTH – Adrenocorticotrophic Hormone

ALDO – Aldosterone

CHOL – Cholesterol

CORT – Cortisol

CRH – Corticotrophin Releasing Hormone/Corticotrophin Releasing Factor

CTS – Corticosterone

DCTS – Deoxycorticosterone

HPGS – 17α-Hydroxyprogesterone

HPNN – 17α-Hydroxypregnenolone

PGS – Progesterone

PNN – Pregnenolone

**References**

1. Walter LM, Auchus RJ. The Molecular Biology, Biochemistry, and Physiology of Human Steroidogenesis and Its Disorders. Endocrine Reviews. 2011;32(1):81–151. Epub November 4, 2010.
