# Supplementary material for: Corticosterone oscillations during mania induction in the lateral hypothalamic kindled rat—Experimental observations and mathematical modeling
Source: PLoS One. 2017 May 18;12(5):e0177551. doi: 10.1371/journal.pone.0177551 (PMC5436765; doi:10.1371/journal.pone.0177551)
Supplement: S1 Table — (DOC) [file pone.0177551.s002.doc]

***Supporting Information PONE-D-15-56451***: Abulseoud *et al.* HPA axis dynamics in LHK rat

**S1 Table. Differential equations describing the temporal dynamics of HPA axis hormones in rodents derived from the reaction model in Table 1 (in the main text).**

$\frac{d\left[CHOL\right]}{dt} = k_{1} - (k_{5}\left[ACTH\right] +k_{18})\left[CHOL\right]$

$\frac{d\left[CRH\right]}{dt} = k_{2}\times D-\left(k_{4}+k_{16}\left[CTS\right]+k_{19}\right)\left[CRH\right]$

$\frac{d\left[ALDO\right]}{dt} = k_{3}+k_{9}\left[CTS\right]-k_{15}\left[ALDO\right]\left[CTS\right]^{2}-k_{22}\left[ALDO\right]$

$\frac{d\left[ACTH\right]}{dt} = k_{4}\left[CRH\right]-\left(k_{5}\left[CHOL\right]+k_{17}\left[CTS\right]+ k_{20}\right)\left[ACTH\right]$

$\frac{d\left[PNN\right]}{dt} = k_{5}\left[ACTH\right]\left[CHOL\right]-(k_{6}+ k_{11}+ k_{24})\left[PNN\right]$

$\frac{d\left[PGS\right]}{dt} = k_{6}\left[PNN\right]-k_{7}\left[PGS\right]-k_{10}\left[PGS\right]-k_{25}\left[PGS\right]$

$\frac{d\left[DCTS\right]}{dt} = k_{7}\left[PGS\right]-(k_{8} + k_{14}\left[CTS\right]^{2})\left[DCTS\right]$

$\begin{matrix} \frac{d\left[CTS\right]}{dt} = k_{8}\left[DCTS\right]-k_{9}\left[CTS\right]+k_{14}\left[DCTS\right]\left[CTS\right]^{2}- k_{15}\left[ALDO\right]\left[CTS\right]^{2}- \\ - k_{16}\left[CRH\right]\left[CTS\right]- k_{17}\left[ACTH\right]\left[CTS\right] - k_{21}\left[CTS\right] \end{matrix}$

$\frac{d\left[HPGS\right]}{dt} = k_{10}\left[PGS\right]+k_{12}\left[HPNN\right]- k_{13}\left[HPGS\right]$

$\frac{\text{d}\left[\text{CORT}\right]}{\text{dt}} \text{=} \text{k}_{13}\left[\text{HPGS}\right] \text{- k}_{23}\left[\text{CORT}\right]$

$\frac{d\left[HPNN\right]}{dt} = k_{11}\left[PNN\right]-k_{12}\left[HPNN\right]$
