# Supplementary material for: Corticosterone oscillations during mania induction in the lateral hypothalamic kindled rat—Experimental observations and mathematical modeling
Source: PLoS One. 2017 May 18;12(5):e0177551. doi: 10.1371/journal.pone.0177551 (PMC5436765; doi:10.1371/journal.pone.0177551)
Supplement: S2 Table — (DOC) [file pone.0177551.s003.doc]

***Supporting Information PONE-D-15-56451***: Abulseoud *et al.* HPA axis dynamics in LHK rat

**S2 Table. Normal basal blood levels of HPA axis hormones in rats as compared to values predicted by the model given in Table 1 (in the main text).**

| **Species** | **Reference value / M*** | **Model / M*** | **Reference** |
| --- | --- | --- | --- |
| **Cholesterol** | (9.6 – 22.9)×10^-4^ | 3.41×10^-4^ |  |
| **CRH** | (2.4 ± 0.2)×10^-12^ | (0.3 – 2.0)×10^-12^ |  |
| **ACTH** | 6.6×10^-12^ | (0.2 – 1.7)×10^-12^ |  |
| **Cortisol** | (2.5 – 7.5)×10^-10^ | (1.5 – 8.0)×10^-10^ |  |
| **Aldosterone** | 3×10^-10^ | (0.7 – 2.4)×10^-9^ |  |
| **Pregnenolone** | 7×10^-10^ | (0.4 – 2.5)×10^-9^ |  |
| **Progesterone** | 2×10^-9^ M | (0.8 – 5.0)×10^-9^ |  |
| **17α-hydroxy**  **pregnenolone** | 2.7×10^-8^ M | (0.2 – 1.1)×10^-8^ |  |
| **17α-hydroxy**  **progesterone** | 1.1×10^-9^ M | (0.4 – 2.2)×10^-9^ |  |
| **Corticosterone** | (1.1 – 70.8)×10^-8^ | (0.4 – 3.0)×10^-7^ |  |
| **11**-**Deoxy**  **corticosterone** | 9×10^-9^ M  1.2×10^-8^ M | (1.3 – 6.8)×10^-7^ |  |

* Concentrations are expressed in moles *per* cubic decimeter, mol dm^-3^ = M.

**References**

1. Giknis MLA, Clifford CB. Clinical Laboratory Parameters for Crl: WI (Han) Rats2008.

2. Yokoe T, Audhya T, Brown C, Hutchinson B, Passarelli J, Hollander CS. Corticotropin-releasing factor levels in the peripheral plasma and hypothalamus of the rat vary in parallel with changes in the pituitary-adrenal axis. Endocrinology. 1988;123(3):1348-54. Epub 1988/09/01.

3. Bocheva A, Dzambazova E, Hadjiolova R, Traikov L, Mincheva R, Bivolarski I. Effect of Tyr-MIF-1 peptides on blood ACTH and corticosterone concentration induced by three experimental models of stress. Autonomic & autacoid pharmacology. 2008;28(4):117-23. Epub 2008/09/19.

4. Thomas M, Northrup SR, Hornsby PJ. Adrenocortical tissue formed by transplantation of normal clones of bovine adrenocortical cells in scid mice replaces the essential functions of the animals' adrenal glands. Nature medicine. 1997;3(9):978-83. Epub 1997/09/01.

5. Eudy RJ, Sahasrabudhe V, Sweeney K, Tugnait M, King-Ahmad A, Near K, et al. The use of plasma aldosterone and urinary sodium to potassium ratio as translatable quantitative biomarkers of mineralocorticoid receptor antagonism. Journal of translational medicine. 2011;9:180. Epub 2011/10/25.

6. Fromm M, Oelkers W, Hegel U. Time course of aldosterone and corticosterone plasma levels in rats during general anaesthesia and abdominal surgery. Pflugers Archiv : European journal of physiology. 1983;399(4):249-54.

7. Punjabi U, Deslypere JP, Verdonck L, Vermeulen A. Androgen and precursor levels in serum and testes of adult rats under basal conditions and after hCG stimulation. Journal of steroid biochemistry. 1983;19(4):1481-90. Epub 1983/10/01.

8. Chen J, Liang Q, Hua H, Wang Y, Luo G, Hu M, et al. Simultaneous determination of 15 steroids in rat blood via gas chromatography-mass spectrometry to evaluate the impact of emasculation on adrenal. Talanta. 2009;80(2):826-32. Epub 2009/10/20.

9. Molteni A, Nickerson PA, Latta J, Brownie AC. Hypertension in rats bearing an adrenocorticotropic hormone-, growth hormone-, and prolactin-secreting tumor (MtTF4). Cancer research. 1972;32(1):114-8. Epub 1972/01/01.
